# Implementation of home-based Digital Rehabilitation in sub-Saharan Africa: a scoping review

**DOI:** 10.1186/s12889-026-30180-8

**Published:** 2026-11-06

**Authors:** Angela Arntz, Mia Vuori, Renée Oltman, Marietta Handgraaf, Franziska Weber, Emmanuel Ngwakongnwi, Matthew Ames, Nassib Tawa, Caroline Mumbi, Kaisa Jokinen, David Tumusiime, Juliette Gasana, Gerard Urimubenshi, Nancy Amaya, Naomi Kingau, Said Khamis Juma, Abdulrahman Khamis, Sarah Harry Mkenda, Asteria Lawrence Menuka, Christian Grüneberg

**Affiliations:** 1 Department of Nursing, Midwifery and Therapeutic Sciences, Bochum University of Applied Sciences, Bochum, Germany; 2 Institute of Rehabilitation, Jamk University of Applied Sciences, Jyväskylä, Finland; 3 Department of Rehabilitation, Physiotherapy Science & Sports, UMC Utrecht Brain Center, University Medical Center Utrecht, Utrecht University, Utrecht, The Netherlands; 4 Institute of Global Health Equity Research, University of Global Health Equity, Kigali, Rwanda; 5 Department of Humanities & Social Sciences, American University of Ras Al Khaimah, Ras Al Khaimah, United Arab Emirates; 6 Department of Rehabilitation Sciences, College of Health Sciences, Jomo Kenyatta University of Agriculture and Technology, Kiambu County, Kenya; 7 Centre of Excellence in Biomedical Engineering and eHealth (CEBE), College of Medicine and Health Sciences, University of Rwanda, Kigali, Rwanda; 8 Department of Physiotherapy, School of Health Sciences, College of Medicine and Health Sciences, University of Rwanda, Kigali, Rwanda; 9 Department of Orthopedics and Rehabilitation, Moi University College of Health Sciences, Eldoret, Kenya; 10 Department of Early Childhood, Primary, Inclusive and Special Needs Education, School of Education, State University of Zanzibar, Zanzibar, Tanzania; 11 Department of Occupational Therapy, School of rehabilitation medicine, KCMC University, Moshi, Tanzania; 12 Department of Prosthetics and Orthotics, School of rehabilitation medicine, KCMC University, Moshi, Tanzania; 13 Institute of Medical Engineering and Mechatronics, Ulm University of Applied Sciences, Ulm, Germany

**Keywords:** Digital Rehabilitation, Digital technologies, Digital intervention, Digital health, Home-based rehabilitation, Remote rehabilitation, Sub-Saharan Africa, Implementation sciences, Scoping review

## Abstract

**Background:**

The need for rehabilitation in sub-Saharan Africa is largely unmet due to challenges such as limited access to rehabilitation, lack of healthcare professionals, high costs, challenging geographical distance and poor health infrastructure. Home-based Digital Rehabilitation may offer an opportunity to overcome some challenges. However, little is known about successful implementation of home-based Digital Rehabilitation in sub-Saharan Africa. We aimed to identify the technologies used and the important factors considered for the implementation of home-based Digital Rehabilitation.

**Methods:**

We performed a scoping review according to the framework proposed by Arksey and O’Malley, with improvements made by Levac et al. The search strategy was developed on PubMed and translated to Embase, CINAHL, Cochrane, Web of science, IEEE, Science Direct and SpringerLink to identify literature published from 2018 to 2023 conducted in sub-Saharan African countries. We performed a gray literature search to supplement the results. We extracted data on technology and implementation determinants based on the domains of the Consolidated Framework for Implementation Research 2.0 (CFIR) and summarized them narratively. In addition, 11 key stakeholders were consulted to explore their experiences with digital technologies and their perspectives on factors relevant to the implementation of home-based Digital Rehabilitation.

**Results:**

We included 25 studies in the final set of papers. Technologies such as applications, web-based programs, videoconferencing, short-message-services, television/radio, social media, and 3-D printers are already utilized for rehabilitation in sub-Saharan Africa. Important implementation determinants were identified in three of five CFIR domains, including intervention effectiveness, relative advantages, costs and the design, client acceptability, the use of implementation frameworks, and appropriate implementation strategies. Barriers to and facilitators of implementation were reported across studies, including the need for sufficient technical infrastructure and ongoing technical support to operate the technology.

**Conclusion:**

The results of this study highlight the need to develop and implement sustainable Digital Rehabilitation approaches that consider all domains of the CFIR 2.0, including structural characteristics, religious and cultural factors, local conditions, local policies and legislation, and intervention financing. The implementation of home-based Digital Rehabilitation requires systemic adaptations, such as the development of an appropriate technical infrastructure and reimbursement mechanism, as well as increasing (digital) health literacy in society.

**Clinical trial number:**

Not applicable.

**Supplementary Information:**

The online version contains supplementary material available at https://doi.org/10.1186/s12889-026-30180-8.

## Background

Globally, approximately 1.3 billion people live with some form of disability, constituting around 16% of the world’s population [1]. Notably, about 80% of these people reside in developing countries, including sub-Saharan Africa (SSA). The SSA region, comprising 49 countries located in the southern part of the African continent, had a total population of approximately 1.18 billion in 2021 [2]. Due to the rapid population growth, the per capita income in SSA is increasing at a slower rate than the gross domestic product (GDP), resulting in the SSA countries ranking lowest in global GDP comparison [3].

Rehabilitation is an essential component of care for people with disabilities to enable them to be as independent as possible in their daily lives and to participate in education, work, leisure and meaningful activities [4]. Currently, the need for rehabilitation is largely unmet. In some low- and middle-income countries, more than 50% of people do not receive the rehabilitation services they need [4].

The sustainable development goals introduced by the United Nations aim to promote prosperity while protecting the planet worldwide. The goals are linked to an action plan including strategies that build economic growth and address a range of social needs including education, health, social protection, and job opportunities, while tackling climate change and environmental protection.

The third sustainable development goal is to “ensure healthy lives and promote well-being for all at all age“ [5]. Thus, rehabilitation coverage worldwide is an essential part that contributes to achieving the goal.

The high percentage of unmet rehabilitation needs in low- and middle-income countries, particularly in the SSA region, is attributable to multiple structural and systemic factors. While Sub-Saharan African countries differ significantly in financing, governance, and the integration of rehabilitation services, many follow a decentralized, tiered health system, with services organized across primary, secondary, and tertiary levels [6]—as observed in countries like Tanzania [7], Kenya [8], Rwanda [9], Ethiopia [10], and Uganda. This structure directly influences the availability and accessibility of rehabilitation services across the region [6, 11]. At the top of this pyramid are tertiary facilities, central and university hospitals, located in urban centers, offering specialized services but remaining largely inaccessible to rural populations. Secondary care is delivered through regional and district hospitals, which are more widely distributed but still scarce, often lacking adequately staffed and equipped rehabilitation departments. Primary care, forming the base of the pyramid, includes health centers and posts in rural and peri-urban communities, where basic medical care is available but rehabilitation is almost entirely absent [12]. Community health workers play a key role in service delivery at this level, but they lack training in rehabilitation [13]. Theoretically, referrals flow upward through this system, from the community level to tertiary hospitals, but in practice, these pathways are often fragmented due to logistical, financial, and infrastructural barriers. For many patients, the pathway to rehabilitation care involves initial contact at the primary level, but due to limited services and referral inefficiencies, most either seek care directly at higher-level facilities or rely on informal, home-based support provided by family members. Consequently, patients either bypass lower levels or remain untreated. Home-based care is widespread [13] but informal, typically provided by community health workers [13] and/or family members [14, 15] without professional training.

As a result of these structural and systemic limitations, access to rehabilitation services across SSA remains constrained. Barriers include a shortage of health professionals [16], high financial costs associated with rehabilitation, inadequate transportation infrastructure [17–19], and significant geographical distances [20]. Additionally, the lack of privacy in rehabilitation centers and fear of stigma associated with accessing healthcare services further reduce the uptake of rehabilitation [18]. Moreover, the scarcity of dedicated rehabilitation infrastructure—particularly at district level—combined with poor referral mechanisms and a lack of follow-up, continues to limit access to essential rehabilitation services [17, 20]. Compounding these issues, communicable diseases remain the leading cause of health loss in SSA, although the burden of non-communicable diseases has also risen in recent years [21]. Many conditions resulting from these diseases necessitate long-term, or even lifelong, rehabilitation. However, the overburdened health systems in the SSA region are seldom equipped to meet these extensive rehabilitation needs. Given the limited resources of health systems in SSA, population aging, and the increasing prevalence of non-communicable diseases, the number of people who would benefit from rehabilitation is rising. Thus, adopting new ways of providing rehabilitation services in SSA is essential to ensure that the demand for rehabilitation is met and to fulfil the United Nations goal of promoting well-being for all [5].

Incorporating digital solutions into home-based rehabilitation (Digital Rehabilitation) presents a promising approach to addressing some of these needs. Digital Rehabilitation is an umbrella term that encompasses the use of digital technologies throughout the rehabilitation process. Its goal is to optimize functioning and reduce disability by interacting with the environment. This includes areas such as prevention, assessment/diagnosis, intervention, monitoring, and evaluation. This approach includes, but is not limited to, the use of tele- and remote rehabilitation applications and services, automated services, robotic technologies, wearables, email, video, voice and SMS (short-message-services) solutions. Digital Rehabilitation is closely related to the empowerment of individuals and communities [22, 23].

Digital health has become an essential component of healthcare transformation across the SSA region, particularly in low-resource settings. Recent estimates suggest that over 1,000 mobile health (mHealth) services are currently active in low-income countries, delivering health information and diagnostic support [24]. The rapid expansion of mobile technology infrastructure has been a key driver of this development. Between 2015 and 2022, the number of smartphone users in SSA rose from 118.1 million to a projected 414.77 million [25]. By 2021, 83% of the population lived in areas covered by mobile broadband networks, and 22% were actively using mobile internet services—with usage rates reaching 40% among adults over 18 years [26]. This increased digital connectivity has enabled the proliferation of digital health interventions across a range of health system functions. A recent scoping review [27] highlights that most digital initiatives aim to strengthen service delivery and improve the efficiency, accessibility, and quality of clinical care. Additional applications include workforce development through training and clinical decision support tools, enhanced health information systems (e.g., electronic medical records), and digital supply chain management for medicines and vaccines. While less common, digital technologies are also being used to support health financing and governance functions [27]. These interventions typically target healthcare providers but also engage clients, system managers, and data platforms, demonstrating the broad applicability of digital health solutions across the health system [27].

The application of home-based Digital Rehabilitation in low-resource settings could facilitate access to rehabilitation [28], overcome transportation issues in reaching professional rehabilitation services [29], and reduce stigma by providing anonymous health services. Digital home-based rehabilitation could also help health systems deliver care at a lower cost [28], thereby optimizing limited resources, including health professional shortages, especially in rural areas. In addition, home-based Digital Rehabilitation can provide care over an extended period of time and increase motivation for clients and healthcare professionals [30].

Despite the promising conditions and benefits of Digital Rehabilitation for enhancing accessibility, the implementation of home-based Digital Rehabilitation in SSA remains limited. One contributing factor is the lack of comprehensive data to support the full-scale implementation of digital health services, considering the specific needs and context of the SSA region. While global guidelines for the implementation of digital health services exist [31], there is little information on what constitutes successful implementation within the SSA context. The region is likely to use different technologies and encounter distinct implementation challenges compared to the global north due to variations in culture, resources, and infrastructure. Therefore, it is crucial to assess the current literature to identify the technologies being used for home-based Digital Rehabilitation in SSA and the factors influencing their implementation. Obtaining such data will provide essential insights for the optimal implementation of home-based Digital Rehabilitation, aiming to ensure equitable access to rehabilitation services for all.

Thus, the aims of this study are (1) to identify the technologies that are currently used for home-based Digital Rehabilitation in the SSA region and (2) to evaluate the important factors for the implementation of home-based Digital Rehabilitation in the SSA region.

## Methods

A scoping review was performed according to the framework proposed by Arksey and O’Malley (2005) [32] and further developed by Levac and colleagues (2010) [33]. The review was carried out between June 2023 and June 2024. Although quality assessment of included studies is not mandatory under the framework proposed by Arksey and O’Malley (2005) [32] and Levac and colleagues (2010) [33], we conducted a quality appraisal of the included studies. We used the PRISMA-ScR (Preferred Reporting Items for Systematic Reviews and Meta-Analyses extension for Scoping Reviews [34]) for reporting (see appendix 1). The registration of this scoping review was published via Open Science Framework (OSF) [35].

### Ethical considerations

The stakeholder consultation was conducted as part of the RADIC project. Before participation, all stakeholders received information about the purpose of the consultation, the project objectives, and how their contributions would be documented and used. Participation was voluntary, and all stakeholders provided written informed consent to participate in the consultation and to allow their contributions to be incorporated into this study. The consultation was conducted in accordance with the ethical principles of the Declaration of Helsinki, including respect for participants, voluntary participation, informed consent, and confidentiality [36].

### Step 1: Identification of Research Questions

The overarching research questions guiding this review were:

- Research question 1 (RQ1): Which technologies are used in the sub-Saharan region for home-based Digital Rehabilitation?
- Research question 2 (RQ2): Which key factors are essential for the successful implementation of home-based Digital Rehabilitation in the sub-Saharan region within the domains of the CFIR 2.0?

### Step 2: Study Identification

To identify relevant studies, we searched in PubMed, Embase, CINAHL, Cochrane, Web of Science, IEEE Xplore, Science Direct, and SpringerLink for published studies. We conducted an initial limited search to identify keywords for the full search strategy. This aimed to find articles about digital technologies used for home-based Digital Rehabilitation and factors important to the implementation of home-based Digital Rehabilitation in SSA. Based on this, we (AA, CG) developed the search strategy for PubMed then expanded it for Embase, CINAHL, Cochrane, Web of Science, IEEE, Science Direct and SpringerLink. The keywords, their synonyms and related terms of “digital technology”, “rehabilitation”, and “sub-Saharan Africa” were used for the search strategy. To ensure comprehensive regional coverage, we explicitly included all countries classified under the SSA region as individual search terms (e.g., Nigeria, Kenya, Ethiopia, Mozambique, etc.). This approach guaranteed that studies from all SSA countries could be identified.

The full search strategy for PubMed is presented in appendix 2. Studies published between January 1, 2018, and July 7, 2023, were eligible for inclusion. The literature search was conducted on July 7, 2023; therefore, studies published after this date could not be considered. This timeframe was selected to focus the review on recent developments in Digital Rehabilitation and on contemporary technologies and implementation contexts in sub-Saharan Africa. Our aim was to capture the current state of implementation and practice. Including older studies may have increased the likelihood of identifying technologies or implementation contexts that are no longer representative of current practice, thereby limiting the applicability of the findings to present-day settings. To complement the findings to the research questions and to minimize the risk of publication bias, we conducted a gray literature search. This included materials in English, French or the native languages by the member institutes of the project “Rehabilitation for all through Digital Innovations and Competencies (RADIC)”, namely English, French, Swahili, and Arabic. We searched gray literature using Google, Google Scholar, Yahoo, openMD, MedNar, Base and specific websites from each country’s government, thesis repositories, and university library websites that offer a comprehensive list of gray literature databases.

### Step 3: Study Selection

#### Eligibility criteria

To select relevant studies, we used the following eligibility criteria (Table 1).

**Table 1:** Eligibility criteria for the scoping review

	Inclusion Criteria	Exclusion Criteria
Type of Publication	*(R)*CT’s, case studies, cohort studies, qualitative studies, mixed-methods	All types of reviews, conference proceedings, study protocols/registrations, commentaries, editorials, abstracts only
Timeframe	2018 – Today (7/7/2023)	Before 2018
Region	Study is conducted in the SSA region	Study conducted outside the SSA region
Digital technologies	All references are included focusing on all types of *digital* technologies (e.g., text messaging, videoconferencing like zoom/skype, applications, robots, gaming via a digital source, virtual/augmented reality, serious gaming, exergaming…)	Technology refers to laboratory or clinics only
Setting	The intervention is conducted at home or in the lifespace such as school or retirement home	The intervention is conducted in the hospital, clinic or rehabilitation center
Intervention	Study describes an intervention intended for a client; intervention refers to rehabilitation based on the definition of the WHO [23]	Invasive procedure (e.g., surgery) performed before using the digital technology, such as in implantable device/sensors; the digital component of the described intervention includes telephone calls or E-Mail only
Implementation	At least one implementation determinant of the CFIR 2.0 [37] should be reported/measured/assessed; intervention described in the study is already implemented	Intervention described in the study is not implemented yet
Condition	No restriction	
Language	English, French, Swahili, Arabic	

We included studies when the digital interventions described were already implemented in one of the countries of the SSA region. For example, we excluded studies, if they measured attitudes towards digital interventions without having participated in such interventions. In addition, we only included studies that measured, described, or assessed at least one implementation determinant. An implementation determinant is a factor that influences the implementation process of an intervention. These determinants can act as facilitators or barriers and exist at multiple levels, including the individual, organizational, community and system levels. To extract relevant information about implementation, we used the Consolidated Framework for Implementation Research (CFIR) 2.0, developed and updated by Damschroder et al. (2022). The CFIR is a determinant framework consisting of implementation determinants on the following five domains: Innovation, Individual, Inner Setting, Outer Setting and Implementation Process. All areas must be considered for effective implementation [37]. We limited our literature search to publications in English, French, Swahili, and Arabic, acknowledging that other languages, such as Portuguese or local languages, are also spoken in SSA. However, none of the team members had sufficient proficiency in additional languages (e.g., Portuguese) to reliably assess and extract data from full-text articles. Including studies in languages beyond our linguistic expertise could have introduced bias or compromised the consistency and methodological rigor of data extraction. To address potential omissions, we conducted an additional search on May 5, 2025, using our full search strategy across all databases, with no language restrictions. This search yielded no eligible publications in languages other than English, French, Swahili, or Arabic.

#### Selection process

We used the web-based software, Covidence (version 2021; Veritas Health Innovation) for study selection. The software removed the duplicates automatically. After a training period, three researchers (AA, CG, MV) independently screened the records according to the eligibility criteria presented in Table 1. First, titles and abstracts were screened and subsequently, full texts were assessed. For every reference, two researchers (AA, MV) individually assessed the reference in terms of these criteria. In cases of disagreement, a third researcher (CG) was included to resolve conflicts.

### Step 4: Data Extraction

The research team developed a data extraction sheet to extract relevant data regarding the research questions, which is provided in Table 2. After a test, two independent researchers (AA, MV) performed data extraction. Disagreements were resolved through discussions and by a third researcher (CG) until consensus was reached.

**Table 2:** Data Extraction Sheet for the scoping review

General information	
	• Title
	• Authors
	• Year of publication • Journal
	• Type of article/study
	• Purpose/aim of the article/study
	• Population’s condition
	• Country where the study was conducted
	• Environment/setting where the technology was used e.g., @home, elderly home, school…
	• Description of intervention including mode of intervention
	• Intervention group
	• Control group
	• Outcome measures
	• Conclusion
Information regarding RQ 1	
	• Technology
	• Name of technology, if information is given
	• Type of technology (short description)
Information regarding RQ 2	
	• Assessed/evaluated/described implementation determinant in the domains of the CFIR 2.0
	• Results regarding the implementation determinant

### Step 5: Stakeholder Involvement

To provide a more comprehensive overview of the technologies implemented in SSA and the factors influencing their implementation, we complemented the literature review findings with input from relevant stakeholders.

Stakeholders were selected using purposive sampling. Selection aimed to include participants with professional expertise in healthcare, social care, digital education, research, or related fields, as well as knowledge of the healthcare and rehabilitation context in sub-Saharan Africa. We deliberately sought variation in professional backgrounds, geographical perspectives, and prior experience with Digital Rehabilitation by including participants both with and without such experience.

The stakeholders were members of the RADIC consortium and were recruited through the consortium’s institutional network. They were affiliated with Jamk University of Applied Sciences, Finland (*n* = 2); the University of Rwanda, Rwanda (*n* = 4); Kilimanjaro Christian Medical University College, Tanzania (*n* = 1); Moi University, Kenya (*n* = 2); and Jomo Kenyatta University of Agriculture and Technology, Kenya (*n* = 2).

The stakeholder involvement aimed to supplement the literature findings by gathering additional insights on the technologies currently used in rehabilitation, their relevance, and key factors influencing the implementation of home-based Digital Rehabilitation in the SSA region. For this purpose, we organized a face-to-face stakeholder meeting with eleven participants (*n* = 11; 100%). The participants included healthcare professionals (*n* = 8; 73%), a social worker (*n* = 1; 9%), an e-learning specialist (*n* = 1; 9%), and a statistical analyst (*n* = 1; 9%). The majority had prior experience in Digital Rehabilitation (*n* = 7; 63%). The characteristics of the participants are presented in Table 3.

**Table 3:** Characteristics of participants from stakeholder meeting (*N* = 11)

Characteristics	Value *n*
Participants’ residence or origin *n* (%)	
Finland	1 (9.1)
Ethiopia	1 (9.1)
Rwanda	4 (36.35)
Tanzania	1 (9.1)
Kenya	4 (36.35)
Age (years), mean (min/max)	43 (27/57)
Sex, n (%)	
Male	4 (36.35)
Female	7 (63.65)
Profession, n (%)	
Physical therapist	6 (54.5)
Occupational therapist	2 (18.2)
Social worker	1 (9.1)
E-learning designer	1 (9.1)
Statistical analyst	1 (9.1)
Current occupation, n (%)	
Researcher	4 (36.35)
Professor/teacher in health sciences	7 (63.65)
E-learning manager	1 (9.1)
Project specialist	1 (9.1)
Prior experience in Digital Rehabilitation, n (%)	7 (63.6)

Note: Multiple responses were possible for current occupation, therefore, the number of responses exceeds the total number of participants

The meeting began with a presentation of the preliminary review results. Stakeholders were then invited to contribute by identifying additional technologies implemented in SSA, assessing the relevance of the identified technologies, and discussing the health conditions targeted by these digital interventions. Furthermore, they shared their perspectives on barriers and facilitators affecting the implementation of home-based Digital Rehabilitation.

All stakeholder contributions were systematically documented in meeting minutes structured according to applied technologies, their relevance, targeted health conditions, and implementation barriers and facilitators. The minutes were sent to participants for verification of accuracy. Subsequently, the minutes were summarized in a report addressing the discussed aspects. The input obtained from stakeholders was integrated into the review findings to enhance the comprehensiveness and contextual relevance of the study. Stakeholder involvement took place after the initial literature analysis and served as an important step to validate and complement the emerging results. The contributions made by the stakeholders are described in the Results section.

### Critical appraisal and risk of bias assessment

Two reviewers (AA, MV) independently performed the risk of bias assessment for RCT’s. They also conducted the quality appraisal for qualitative studies and mixed-method studies. For this they used the revised Cochrane Risk of Bias 2.0 tool for randomized trials (RoB 2.0) [38], Critical Appraisal Checklist for Qualitative Research by the Joanna Briggs Institute [39], and the Mixed Methods Appraisal Tool (MMAT) Version 2018 [40]. We resolved any disagreements between two review authors by discussion and, if necessary, by consultation with the wider review team. No studies were excluded from the review based on their methodological quality. Appraisal of the studies included was still undertaken to assist with interpretation of their findings. The results are summarized in appendix 3.

### Step 6: Summarizing Results

The extracted data were summarized and presented in tabular form. Absolute frequencies and percentages were calculated manually using a calculator to describe the distribution of study characteristics, technologies used, and implementation determinants across the included studies. The findings were subsequently synthesized narratively according to the two RQs. Implementation determinants were categorized according to the domains of the CFIR: Innovation, Individual, Inner Setting, Outer Setting, and Implementation process. Where relevant, statistical results reported in the original studies were reproduced without recalculation, pooling or additional statistical analysis.

## Results

**Fig. 1:**
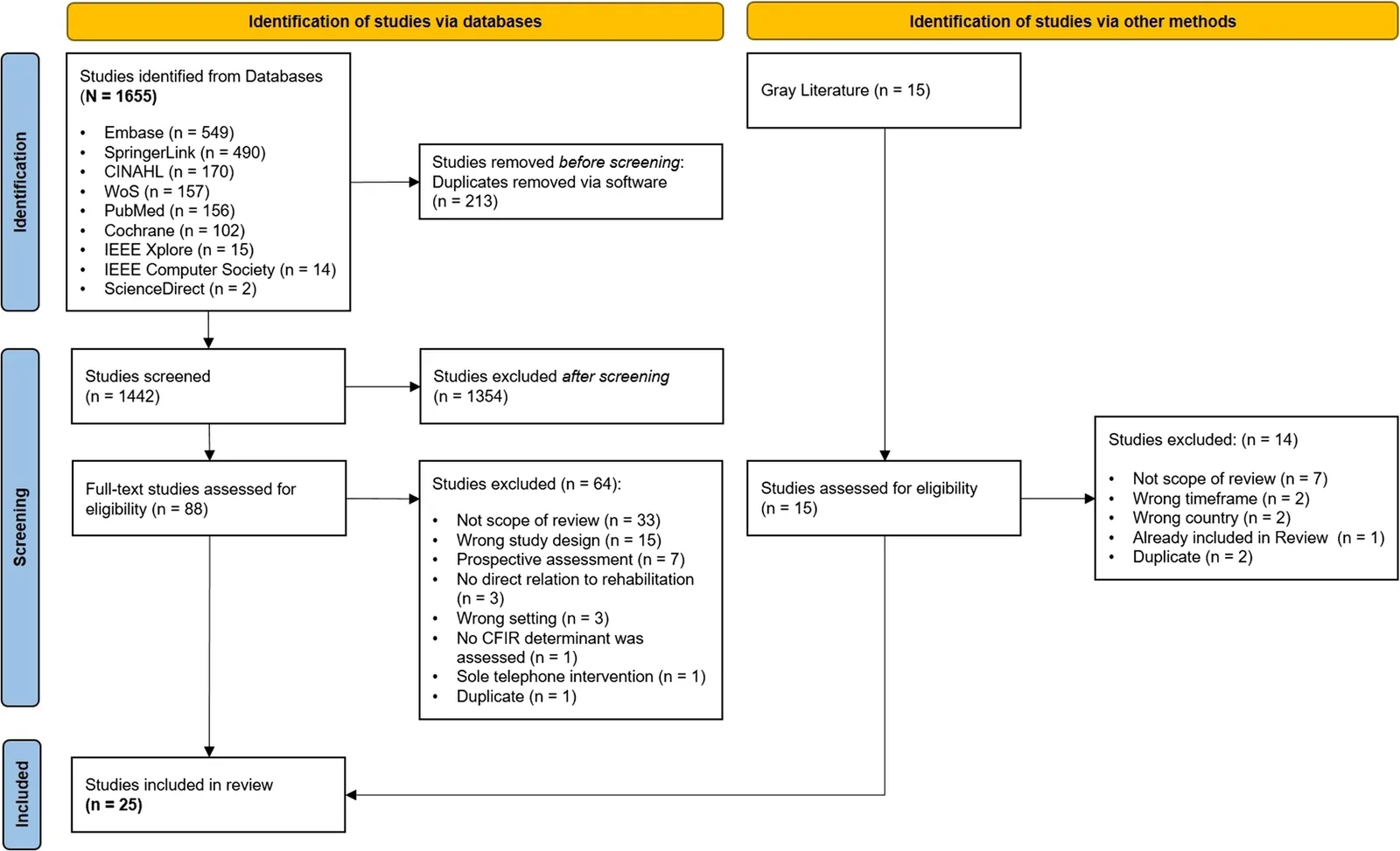
Paper selection process

The study selection process is presented in Fig. 1.

### Study characteristics

We found 1655 articles based on our search strategy. After applying the eligibility criteria, the final set of studies consisted of 25 studies including 24 studies from scientific database searches and one via gray literature search. The study set contained randomized controlled trials (RCT’s) (*n* = 10; 40%), mixed-method studies (*n* = 4; 16%), pilot studies (*n* = 4; 16%), qualitative studies (*n* = 2; 8%), cohort studies (*n* = 2; 8%), a process evaluation study (*n* = 1; 4%), a cross-sectional study (*n* = 1; 4%), and a non-randomized trial (*n* = 1; 4%).

The two most represented health conditions were Human Immunodeficiency Virus (HIV) (*n* = 6; 24%) and depressive symptoms/depressions (*n* = 5; 20%), followed by stroke (*n* = 3; 12%), hearing disorders/loss (*n* = 2; 8%), and diabetes (*n* = 2; 8%) in the included studies. All other conditions appeared once in the study set: chronic low back pain (*n* = 1; 4%), transtibial amputation (*n* = 1; 4%), malaria (*n* = 1; 4%), tuberculosis (*n* = 1; 4%), maladaptive cognition (*n* = 1; 4%), and burnout (*n* = 1; 4%). In one study the target population was described as “clients receiving occupational therapy services” and was therefore not further specified (*n* = 1; 4%).

Most of the studies were conducted in South Africa (*n* = 9; 36%), Nigeria (*n* = 6; 24%) and Kenya (*n* = 6; 24%). Further study characteristics are reported in Table 4.

**Table 4:** Characteristics of the included studies

Authors and Year	Design	Country	Population’s condition	Type (name of technology) in intervention arm	Content digital intervention	Implementation determinant/construct of the CFIR 2.0	Results of implementation determinant
Abiodun et al. (2023) [41]	Cross-sectional study	Rwanda	Not specified. People receiving occupational therapy services.	Text-messaging (SMS) or App (WhatsApp) or telephone calls/ App alone or in combination with face-to-face session or telephone calls	Providing activity guidelines and specific techniques to facilitate activities.	Individual/Motivation	Motivation: majority of clients were moderately satisfied with the sessions (66.7%) and the rest (33.3%) were highly satisfied with the sessions.
Adewuya et al. (2019) [42]	Pilot study	Nigeria	People with depression aged between 18–60 years	Text- messaging (SMS) + usual care (medication and face-to-face sessions)	Reminders for clinic, psychological sessions, medication intake, and overall adherence to treatment protocols.	Innovation/Relative advantage, Individuals/ Motivation	Relative advantages: higher adherence to treatment, recovery rate, quality of life, retention in treatment, and more cost effective compared to control group (usual collaborative stepped care). Motivation: acceptance level of intervention was high.
Boivin et al. (2019) [43]	RCT	Uganda	Children with Malaria aged between 5–12 years	Web-based program (Captain’s Log^®^)	Cognitive training for attention, working memory and nonverbal reasoning.	Innovation/Evidence base, relative advantage	Innovation evidence base, relative advantage: participants receiving intervention were significantly better than the passive control arm on intellectual abilities (composite of all cognitive domains) (*p* = 0.04), sequential processing (working memory) (*p* = 0.02), and the Conceptual Thinking subtest (*p* = 0.02) from the planning global domain, which is a measure of executive function and reasoning.
Chory et al. (2022) [44]	Mixed-methods study	Kenya	Youths with HIV aged between 10–19 years	App (WhatsApp^®^)	Counseling and peer support on stress management, drug and alcohol abuse prevention, intimate relationships, adherence, disclosure, and stigma.	Innovation/Relative advantage; Individuals/Need, motivation	Relative advantages of intervention: increase of treatment adherence, stigma reduction, and mental and behavioral health. Need, motivation: participants reported overall positive experiences and indicated that the platform encouraged peer network development.
Chory et al. (2021) [45]	Qualitative study	Kenya	Youths with HIV aged between 9–19 years	App (WhatsApp^®^)	Peer support intervention including positive support between members, weekly group discussion topics, answering participants’ questions.	Individual/Need, Motivation; Implementation Process/Assessing context	Need, Motivation: participants reported overall positive experiences. They endorsed potential benefits for treatment adherence, stigma reduction, and mental and behavioral health. All participants supported intervention expansion. Assessing context: participants identified household and school responsibilities as barriers to active participation, as well as logistical issues including power outages, insufficient phone credit, internet problems, and learning to use the mobile device. Caregivers were also mentioned by several participants as potential barriers to their participation in the intervention, with some caregivers not understanding why the adolescent was using the phone.
Dulli et al. (2020) [46]	RCT	Nigeria	Adolescents with HIV aged between 15–24 years	Social media (Facebook) + usual care (viral load tests, active case management, and enhanced adherence counseling for patients with unsuppressed viral loads.)	Peer support groups include non-health activities like puzzles, riddles and discussions. Information on key topics about HIV were given and the knowledge was tested by polls.	Innovation/Evidence base; Relative advantage	Innovation evidence base, relative advantage: improved HIV knowledge (*p* = 0.003) and high acceptability are encouraging, despite a lack of measurable effect on retention. Retention was greater than anticipated in both groups. Web-based delivery of support group interventions can permit people to access information and other group members privately, when convenient, and without travel.
Fatoye et al. (2020) [47]	RCT	Nigeria	People with nonspecific chronic low back pain aged between 20–65 years	App (not specified)	Exercise intervention, back care education, performance feedback, and progress tracking.	Innovation/Evidence base, Relative advantage, Costs	Innovation evidence base, relative advantage: there were no significant or clinically relevant differences in disability in the measurements at weeks 4 and 8 between the intervention and control group (*p* > 0.05). Quality-adjusted life years gained were 0.085 for intervention group and 0.084 for the control group. Costs: the mean cost estimates per person were 22,200 naira (US $61.7) in the intervention group and 38,200 naira (US $106) in the control group (Clinic-Based McKenzie Therapy).
Gericke et al. (2021) [48]	Qualitative study	South Africa	Students with symptoms of depression aged between 17–20 years	Web-based platform (ICare)	Cognitive behavioral therapy intervention.	Innovation/Relative advantage; Individuals/Motivation	Relative advantage of innovation: anonymity, privacy and accessibility of intervention was appealing for participants, believing this facilitated use of the intervention and overcame stigma associated with accessing traditional counselling services. Motivation: students also reported disappointment with the lack of human contact and immediate responsiveness, articulating an expectation that the e-intervention would mimic face-to-face psychotherapy.
Ivanova et al. (2019) [49]	Pilot study	Kenya	Adolescents with HIV aged between 15–24 years	Web-based platform (ELIMIKA platform)	Improving HIV/and therapy knowledge, perceived importance of adherence, perceived self-efficacy in adhering and future intentions towards adherence.	Innovation/Relative advantage, Design; Individuals/Need, motivation; Implementation/ Assessing context, Engaging	Relative advantage of intervention: there was not a significant change in knowledge and behavior, but adherence intentions after 3 months intervention period have improved. Design: majority (95%) stated their intentions to use the platform again and 87% would recommend it to others. Need, motivation: participants were satisfied with the main features of the web platform and stated that they would use it again (95%). Assessing context: barriers for implementation were lack of money for internet, no permission to use smartphones in schools, low internet literacy, short implementation time. Engaging: Involving volunteers to support implementation and to help participants in using online platform.
Jarvis et al. (2019) [50]	RCT	South Africa	People ≥ 60 years with maladaptive cognition	App (WhatsApp^®^) + face- to-face session	Individualized messages addressing maladaptive cognitions.	Innovation/Evidence base, Relative advantage	Innovation evidence base, relative advantage: there were significant positive changes in social cognition (*p* = 0.008) and loneliness (*p* = 0.001) levels compared to the control group (wellness program). At 1-month follow-up, even with a significant reduction in WhatsApp usage, a significant reduction in loneliness was maintained.
Khatib & Hlayisi (2022) [51]	Mixed- methods study	South Africa	People with hearing loss aged between 19–59 years	Web-based platform (not specified) + face-to-face sessions	Auditory training.	Innovation/Evidence base, Relative advantage, design, Costs; Individual/Motivation	Innovation evidence base, relative advantage: improvement in perceived hearing aid benefit, and improvement in listening skills. High compliance rate (84.82%) with minimal clinician contact time at 3 h 25 min over 5–6 weeks. Design: all participants indicated that they believed people would need initial technical assistance to be able to learn to use the programme quickly. Costs: minimal program costs at an average of R1350.00 per participant Motivation: participants reported positive experiences.
Manyazewal et al. (2022) [52]	RCT	Ethiopia	People ≥ 18 years with tuberculosis	App (evriMED500^®^) + clinic visit on site	Medication reminder and remote monitoring of medication intake/adherence.	Innovation/Evidence base, relative advantage, costs	Innovation evidence base, relative advantage: the therapy was associated with higher health-related quality of life (*p* < 0.001) compared with standard in-person therapy. Costs: the therapy was associated with lower catastrophic costs compared with standard in-person therapy (*p* < 0.001).
Odetunde et al. (2020) [53]	Pilot study	Nigeria	People with stroke	Web-based program (not specified)	Exercise intervention including demonstrations of task-specific mobility-task and postural training; trunk exercises, and over ground walking.	Innovation/Costs; Individual/Motivation	Costs: participants had prior home access to a video player at no-cost. Motivation: participants adhered to the exercises as recorded. The intervention was acceptable among the participants.
Ofoegbu et al. (2020) [54]	RCT	Nigeria	Students with symptoms of depression aged between 17–41 years	Internet + telephone calls (not specified)	Psychoeducation, interactive peer support, cognitive disputation, behavioral homework assignments, role-play, and depression management.	Innovation/Evidence base, Relative advantage	Innovation evidence base, relative advantage: the intervention was significantly effective in reducing depression among university students in the treatment group compared to those in the usual-care control group (*p*= 0.0005).
Osborn et al. (2020) [55]	RCT	Kenya	Students with depression aged between 13–18 years	Web-based platform (Shamiri-Digital)	Information about growing-mindset, exercise on gratitude, and life-values.	Innovation/Evidence base, Relative advantage	Innovation evidence base, relative advantage: greater reduction in adolescent depression symptoms in both the full sample (*p* = 0.028) and a sub-sample of youths with moderate-to-severe depression symptoms (*p* = 0.010) from baseline to two-week follow-up compared to the control group. There were no significant effects on anxiety symptoms, well-being, or happiness.
Owolabi et al. (2020) [56]	RCT	South Africa	People ≥ 18 years with diabetes	Text-messaging (SMS) + usual care (all medical visits, tests, and diabetes support programs at the clinic)	Therapy reminder aiming at improving adherence of diabetes therapy.	Innovation/Evidence base, relative advantage	Innovation evidence base, relative advantage: no significant difference in medication adherence between intervention and control group (*p* = 0.88). The adjusted mean change in the medication adherence level was not significant (*p* = 0.79). There was, however, a low level of adherence to dietary recommendations, and physical activity at baseline, and both groups demonstrated a non-significant increase in dietary (*p = *0.98) and physical activity (*p* = 0.99) adherence from baseline to the follow-up period.
Pandya (2021) [57]	Cohort study	Kenya, South Africa (Mumbai, Bangkok)	Chaplains with burn-out symptoms and emotional exhaustion	App (M-App)	Providing guided meditation and relaxation techniques	Innovation/Relative advantage	Relative advantage: lesser emotional exhaustion (*p* = 0.01) and depersonalization (*p* = 0.001) as burnout markers and higher personal achievement (*P* = 0 0.001) and resilience (*p* = 0.001) as compared to control group.
Ratanjee-Vanmali et al. (2020) [58]	Process-evaluation study	South Africa	People with hearing loss	Web-based platform (not specified) + face-to-face sessions	Hearing screening test and providing audiological intervention, counseling, and coaching.	Innovation/Relative advantage; Individuals/Motivation	Relative advantage: time-efficient, supporting continuation with hearing healthcare. Motivation: client experiences with this online screening test were positive.
Ratto et al. (2021) [59]	Cohort study	Uganda, Tanzania, (Cambodia)	People with transtibial amputation aged between 5–25 years	3-D printing (3DPA device) + gait training	Decrease the manufacturing time for prosthetic devices and supporting clients in activities of daily life.	Innovation/Relative advantage, Design	Relative advantage: regular devices were rated significantly higher within the ambulation section’s questions for ability to walk, to walk up and down stairs, and of device use per week compared to 3-D printed devices. This resulted in an overall higher ambulation category rating for regular devices. Design: very few 3-D printed devices were reported as loose, damaged, or broken.
Sap et al. (2019) [60]	Non-randomized clinical trial	Cameroon	Adolescents with diabetes aged between 13–26 years	App (WhatsApp^®^)	Education on the knowledge of the disease, diets, and glycemic control.	Innovation/Relative advantage	Relative advantage: increase in knowledge on diabetes (*p* < 0.01) and reduction of acute complications decreased from 28% to 16% (*p* = 0.46). In the short-term, there was no positive effect on glycemic control shown.
Sarfo et al. (2018) [61]	Pilot study	Ghana	People with stroke	App (9zest Stroke App^®^) + telephone conferences	Exercise intervention with comprising of four categories: (1) mobility, upper and lower limb strengthening, (2) dexterity to improve fine motor movements, (3) seated and standing balance exercise and (4) Walking endurance.	Innovation/Relative advantage; Individual/Motivation; Implementation Process/Assessing context	Relative advantage: improvement in the Montreal cognitive assessment scores from a baseline mean value of 18.2 ± 4.3 to 20.4 ± 4.7 at month 1 and 22.2 ± 7.6 ( *p* = 0.047, comparing month 3 with baseline), and a reduction in functional impairment ( *p* = 0.0001). Motivation: a significant majority of the participants expressed satisfaction with this medium of rehabilitation service delivery. Assessing context: internet connectivity negatively affected full compliance with the intervention.
Simpson et al. (2021) [62]	Mixed-method study; process evaluation study	Zambia	Adolescent pregnant woman with HIV	App (Rocket.Chat^®^)	Counseling and peer support on social help, relationships, stigma, HIV, and medication adherence.	Individuals/Need, Opportunity, Motivation, Implementation process/Implementation facilitators	Need, opportunity, motivation: anonymity made it easy for participants to communicate openly and reduce the burden of stigma. Implementation facilitators: peer-led structure of the social support groups was an important factor as it encouraged a democratic and intimidating space for women.
Teriö et al. (2019) [24]	Mixed-methods study	Uganda	People with stroke	Text-messaging (F@ce™) + face-to-face sessions	Reminder to improve physical activity, scoring of the performance of the client’s activity and motivational messages.	Implementation process/Assessing Context, Reflecting and evaluation	Assessing context: mechanisms contributing to the implementation of the intervention included engaged facilitators and motivated participants. Challenges in client recruitment and poor information dissemination were some of the mechanisms impeding the implementation. Reflecting and evaluation: eight categories emerged that seemed important for the implementation: (1) perceptions on facilitation, (2) using scientific and experience-based knowledge, (3) tailoring the intervention to local need (family centered), (4) supportive working culture, (5) barriers to the service delivery, (6) implementers’ interaction with the intervention, (7) perceptions on motivations and values, and (8) improving the model and enabling sustainability.
Venter et al. (2019) [63]	RCT	South Africa	People ≥ 18 years with HIV	App (SmartLink) + clinic visit on site	Providing laboratory results, appointment reminders, and information about the laboratory tests, therapy adherence, and HIV in general.	Innovation/Evidence base, Relative advantage	Innovation evidence base, relative advantage: linkage to care between 2 weeks and 8 months was 48.6% in the intervention group versus 45.1% in the control (*p* = 0.52) and increased to 64.1% and 61.0% (*p* = 0.55), respectively, after the initial 8-month period. Moreover, youth aged 18 to 30-years showed a statistically significant 20% (*p* = 0.01) increase in linkage to care for the intervention group.
Wasil et al. (2021) [64]	RCT	Kenya	Students with depression aged between 13–18 years	Web-based platform (Shamiri-Digital)	Information about growing-mindset, exercise on gratitude, and life-values.	Innovation/Evidence base, Relative advantage, Costs	Innovation evidence base, relative advantage, costs: intervention costs US $3.5. Depending on the definition of clinically meaningful improvement, 7.1 to 9.7 students needed to receive the intervention for one student to experience a clinically meaningful improvement, which translated to a cost of US $25.35 to US $34.62 per student. Under a worst-case scenario (i.e., assuming the highest treatment cost and the strictest effectiveness definition), the cost to achieve clinically meaningful improvement was US $92.05 per student.

### Research question 1: Which technologies are used in the sub-Saharan region for home-based Digital Rehabilitation?

Five different technologies could be identified among all included studies: mobile applications (apps) [41, 44, 45, 47, 50, 52, 57, 60–63], short-message-services (SMS) [24, 41, 42, 56], web-based programs [43, 48, 49, 51, 53–55, 58, 64], social media [46], and 3D printing [59].

In eleven studies, apps (*n* = 11; 44%) [41, 44, 45, 47, 50, 52, 57, 60–63] and in four studies SMS (*n* = 4; 16%) [14, 34, 39, 40] were used as part of rehabilitation in SSA. Apps were either utilized in conjunction with face-to-face sessions (blended intervention [65, 66]) [41, 50] or as a reminder and monitoring tool [52, 63] besides the regular rehabilitation process. Apps were also used as stand-alone interventions [44, 45, 47, 57, 60–62], with the option of contacting healthcare professionals when necessary. In all studies, SMS were used in conjunction with face-to-face rehabilitation [24, 41, 42, 56].

In ten studies (*n* = 10; 40%) [24, 41, 42, 44, 45, 50, 56, 60, 62], apps and SMS have been used for text messaging. Text messages were applied to provide individualized messages with content aimed at countering maladaptive cognition [50], activity guidelines, activities or specific techniques [41]. Additionally, text messages were used to provide information on the health condition [60], for digital counseling [41, 45, 62] and/or to initiate peer support [44, 45, 62]. In peer support groups people with the same condition come together and exchanged experiences and discussed diagnosis-related topics under the guidance of a facilitator. In these groups, people could share their experiences, motivate and encourage each other. The moderator or invited experts also provided information via those groups.

In three studies (*n* = 3; 12%) an app or SMS was used to remind participants to take their medication, monitor medication adherence [42, 52, 56] and attend therapy sessions [42]. Furthermore, SMS was used to remind the clients of improving physical activity and to keep an activity logbook [24]. Another study digitally supported clients with HIV by providing them with diagnostic results, explanations about the tests and the disease HIV, and sending reminders for appointments via an app [63]. Two studies provided physical exercises (*n* = 2; 8%) [47, 61] and education for the health condition [47] or meditation for people with burnout symptoms [57] via an app.

In nine studies, web-based programs were utilized in rehabilitation (*n* = 9; 36%). Web-based programs were applied for digital peer support [49], as a cognitive intervention for people with depression [48, 54, 55, 64] and children with malaria [43], testing and providing rehabilitation for hearing loss [58] and hearing disorders [51], and providing an exercise intervention for stroke survivors [53].

Social media was used to form peer support groups for young people living with HIV [46]. Those groups were led by a trained facilitator and were hidden from public to create a safe space for the participants. In these groups, non-health activities were conducted like puzzles, riddles and discussions. Information on key topics about HIV were given and the knowledge was tested by polls.

One project carried out in Uganda, Tanzania, and Cambodia [59] moved 3D printers to a rural area where prosthetics were directly printed for people with transtibial amputation almost without waiting time. The stakeholders also consider 3D printing as important for rehabilitation, as it can reduce the cost of producing prostheses.

The stakeholders added television (TV)/radio as another important technology for rehabilitation in SSA. They explained that TV and radio are already used for public health campaigns and for prevention of specific health conditions. The stakeholders underlined that TV and radio coverage is generally widespread in SSA and can reach large populations without requiring access to additional digital technologies. In addition, they cited video conferencing as another digital technology currently being used in home rehabilitation in SSA to deliver interventions remotely, provide digital counseling, and monitor exercise performance.

### Research question 2: Which key factors are essential for the successful implementation of home-based Digital Rehabilitation in the sub-Saharan region within the domains of the CFIR 2.0?

Three domains of the CFIR 2.0 [37] were assessed in all included studies, namely the domains Innovation, Individual, and Implementation Process. No information could be extracted regarding the domains Inner Setting and Outer Setting.

#### Innovation

The domain Innovation encompasses all factors pertinent to the intervention being implemented. This domain was evaluated in 21 studies (84%). Within this domain, key aspects assessed included the effectiveness (“Innovation evidence base”), relative advantages, costs and the design of the intervention.

The results show that apps were associated with improvements in quality of life among people with tuberculosis [52], increase social cognition and decrease the feeling of loneliness among people with maladaptive cognition [50], and increase the functionality among people with chronic low back pain [47]. Venter et al. [63] also showed that an app improves the linkage to care for people with HIV by providing laboratory results, appointment reminders, and information about the laboratory tests, therapy adherence, and HIV in general.

Peer support groups delivered via the internet are effective in decreasing depression symptoms in students [54] and in increasing disease knowledge in adolescents with HIV [46].

Web-based interventions for younger adults lead to a greater reduction of depression symptoms [55, 64]. Web based interventions are also effective for children with malaria to improve cognitive abilities, sequential processing, functioning, and reasoning compared to a passive control group [43].

One RCT showed no effectiveness of a digital intervention: SMS seems not to be effective in increasing medication adherence and providing dietary recommendations or increasing physical activity in people with diabetes [56]. However, Adewuya et al. [42] and Chory et al. [44] demonstrate in a pilot study that text-messaging could have positive effects on treatment adherence in people with depression and HIV. In another pilot study, Sap et al. [60] showed that education on the disease, diets, and glycemic control via text-messaging could affect the knowledge on diabetes, acute complications positively, but have no effect on glycemic control.

Sarfo et al. [61] indicated in a pilot study improvements in cognitive skills and a reduction in functional impairment in people after stroke. A meditation app seems to have some effects on emotional exhaustion and depersonalization as burnout markers and higher personal achievement and resilience as compared to control group who used a leisure app [57].

Other advantages of the digital intervention besides the effectiveness are an increased accessibility [44], continuation of healthcare [58], and retention in treatment [42]. Furthermore, digital interventions could ensure anonymity of the clients and can thus decrease stigmatization [44, 48]. Digital interventions are also time-efficient [58].

The costs of an intervention are also important for the implementation success. Five studies (*n* = 5; 20%) reviewed the costs of the intervention. The studies showed that the implemented intervention is cost-effective in clients with chronic low back pain [47] and for those with depression [42]. In other studies, the intervention was associated with low or minimal costs for clients with depression [64], stroke [53], hearing loss [51]. Additionally, it reduced catastrophic costs for people with tuberculosis [52] compared to the control group. All interventions were transmitted either via an app or via a web-based program.

The usability was tested in web-based programs [49, 51] and in 3-D printed orthosis [59].

#### Individual

The domain Individual highlights the importance of the roles and characteristics of individuals involved with implementing, delivering, and/or receiving the innovation. Eleven studies assessed the domain Individual (*n* = 11; 44%).

In all studies the “innovation recipients”, thus the clients were asked about the experience, satisfaction or acceptability with the digital intervention.

In most studies, clients reported good experiences with the intervention [41, 44, 45, 51, 58, 61, 62]. They felt more motivated to participate in the intervention [45] and to continue the intervention [58]. Clients felt less stigmatized [44, 45, 62] and perceived positive effects on mental and behavioral health [45]. Clients in the study by Chory et al. [44] also noticed that the digital intervention gave them a new platform to discuss issues that they had not been able to discuss before and gave them a new sense of community. In Khatib & Hlayisi’s study, clients experienced an enjoyable training with the digital intervention and felt that they received helpful tips [51]. In addition, the clients rated the interventions as time-saving and valuable [58].

In three studies the clients accepted the intervention [42, 49, 53] and were satisfied with the content, the functions and mode of delivery [41, 49, 58, 61].

Only participants in the study by Gericke et al. [48] reported that the lack of human contact and immediate responsiveness was a negative aspect of the intervention.

#### Implementation process

**Fig. 2:**
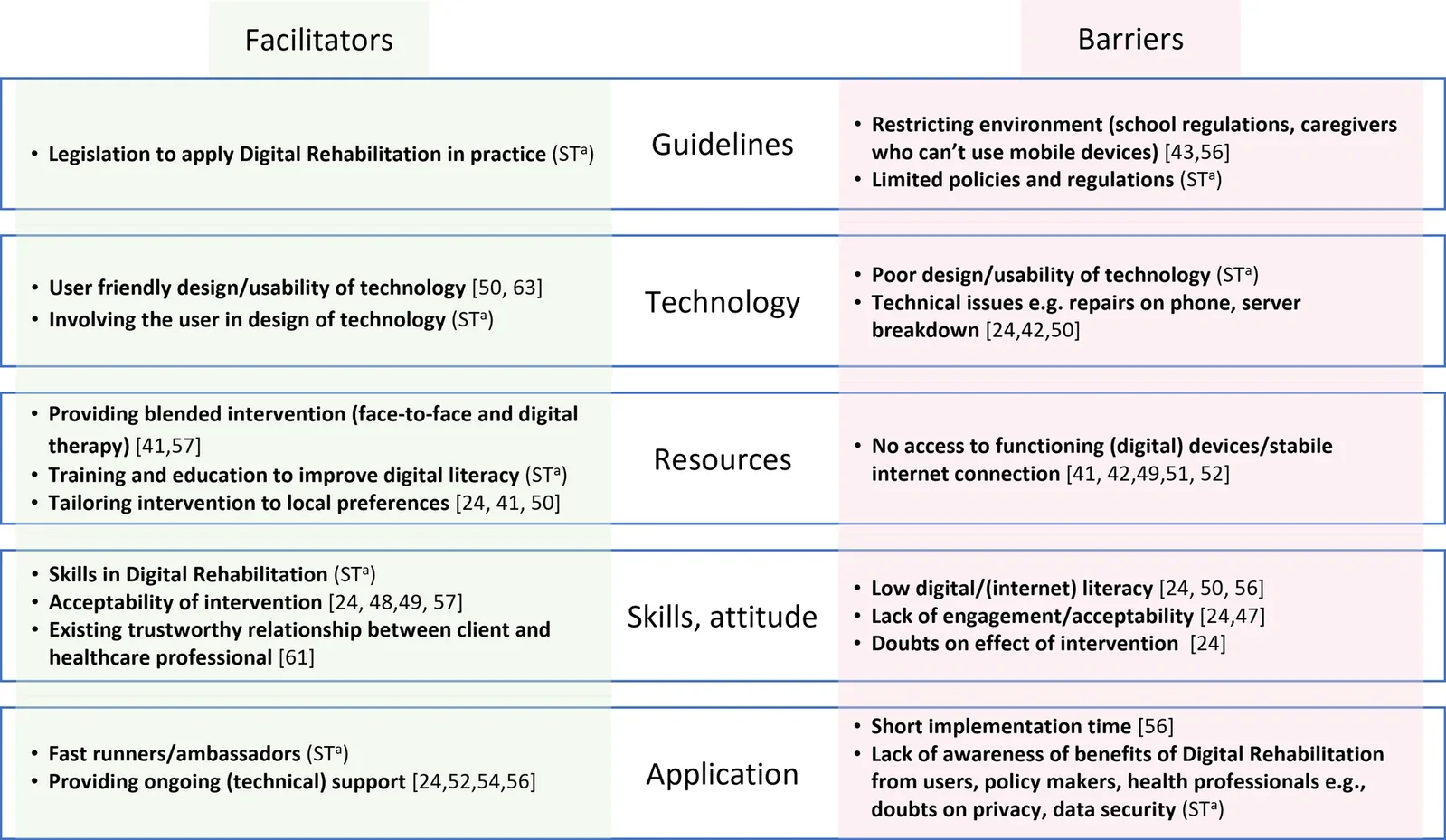
Facilitators and barriers for the implementation of home-based Digital Rehabilitation in SSA. a: This factor is extracted from the stakeholder meeting that was conducted as a part of this scoping review, see step 5 in the methodology

The domain Implementation Process describes the activities and strategies used to implement the innovation successfully. One study used an implementation process framework to guide implementation process [24] (*n* = 1; 2.5%). Twenty studies reported different activities and/or strategies aimed to implement the intervention (*n* = 20; 80%). Fifteen studies reported on facilitators and barriers regarding the implementation (*n* = 15; 16%). They are presented in Fig. 2.

Teriö et al. [24] used The Medical Research Council (MRC) guidance and the Promoting Action on Research Implementation in Health Services (i-PARIHS). The MRC was applied to identify the key processes of the implementation process and the i-PARIHS framework was used to reflect on four dimensions that could influence the implementation outcomes, namely the innovation, recipients, context, and facilitation.

The implementation strategies reported in the included studies were grouped in the categories defined by Waltz et al. [67]. Table 5 depicts the strategies described in the studies categorized according to the nine general categories by Waltz et al. [67].

**Table 5:** Reported implementation strategy categorized by the implementation categories defined by Waltz et al

Implementation category by Waltz et al.	Individual reported strategy and citation
Engage consumers	Providing incentives to participate in the intervention [24,50,59,62] Offering hardware and software needed for intervention [44–47,59,61,62] Increasing demand by starting a public health campaign to inform about the importance of a specific health condition [58]
Use evaluative and iterative strategies	Reflecting and evaluating the success of the implementation [24,58]
Change infrastructure	No results found
Adapt & tailor to the context	Tailoring intervention to the resources, needs and preferences of the users [24,42,46,49,50,53,55–57,60,62,64]
Develop stakeholder interrelationships	No results found
Utilize financial strategies	Providing a robust pricing model for the intervention [58]
Support clinicians	Training for healthcare professionals to deliver digital intervention [24,42]
Provide interactive assistance	(Trained) volunteers, peers or family members [24,46,49,50], project staff [43,61] to support clients in the use and handling of digital technologies
Train and educate stakeholders	Training for clients on the use of the technology [50,52]

The most common implementation strategies involved consumer involvement (*n* = 12; 48%) and adapting the intervention to the context (*n* = 12; 48%).

Providing interactive assistance (*n* = 6, 24%), was also commonly reported. Furthermore, using evaluative and iterative strategies (*n* = 2; 8%), supporting clients (*n* = 2; 8%), as well as training and educating stakeholders (*n* = 2; 8%) were also reported. Most studies included more than one implementation strategy, and/or involving multiple strategies across categories. The strategy “using existing infrastructure“ [28, 30, 41] could not be grouped to a category by Waltz et al.

Client’s internet/digital literacy or literacy overall play an important role [24, 49, 62] in the implementation process. Without sufficient (digital) literacy, individuals may have difficulty navigating through rehabilitation platforms, understanding instructions or communicating effectively with healthcare professionals via digital media. On the one hand, providing technical support to clients is essential for troubleshooting technical issues throughout the rehabilitation process and ensuring smooth operation of Digital Rehabilitation platforms. Ongoing technical support can be provided through dedicated support teams like (trained) volunteers, peers or family members [24, 46, 49, 50] or project staff [43, 61]. On the other hand, clients can be trained before implementing the intervention how to use the device on their own [50, 52].

Even if more people own a smartphone and the internet coverage is becoming better in SSA there are still a large number of people not possessing a smartphone or having internet access. In seven studies [44, 45, 47, 59, 61, 62] researchers equipped clients with smartphones with a preinstalled app and respective internet bundles not to exclude people from the intervention without having a smartphone. Tailoring digital interventions to already implemented infrastructure [49, 52, 55] by delivering digital interventions via computers that are already installed in schools, community centers or hospitals is another strategy to overcome the barrier that not every client can afford a (smart)phone or internet access. Another approach is to adapt the intervention for low-tech solutions like SMS. SMS-based interventions do not require an Internet connection or a smartphone and are also cost-saving [24, 41, 42, 62]. Another strategy is to offer blended interventions, as implemented in six studies [24, 41, 42, 50, 51, 58]. Blended interventions are interventions in which digital components are mixed with face-to-face sessions [65, 66] e.g. in a rehabilitation center/hospital/community. The advantage is that clients do not have to rely exclusively on digital services if, for example, the client lives in a region, where internet coverage is low.

Another important factor is that the intervention should be tailored to the needs of the target group and that the intervention is properly embedded in the local context [24, 41, 62]. For example, an app integrated in rehabilitation should be translated to the local language to ensure the content is usable for people, who do not understand English. Another example is to design a community-based intervention and not client-centered intervention as in high-income countries to meet the local preferences. It leads to a higher degree of fit with existing practices and values, which in turn increases the acceptance of the people involved in the intervention. Stakeholders added that all digital devices and interfaces used in Digital Rehabilitation should be designed to be intuitive and easy to use, including people with various levels of digital skills or disabilities. This may involve incorporating features like voice commands, simple visual cues, and text-to-speech capabilities to enhance accessibility for users with unique needs. For that reason various studies involved clients, family members and healthcare professionals in the development of digital tools or programs to ensure a user-friendly design of the device/program [24, 42, 46, 49, 50, 53], 55– [57, 60, 62, 64].

## Discussion

### Principal findings

This scoping review mapped the digital technologies applied in home-based rehabilitation in SSA and analyzed the factors relevant to their implementation. The findings indicate a diverse range of digital technologies currently employed in rehabilitation practices throughout SSA. These included apps, web-based programs, SMS, social media platforms, and 3D printing. Among these, apps and web-based programs emerged as the most widely implemented technologies. Interestingly, some technologies commonly used in other contexts like videoconferencing [22] were not found in the literature for rehabilitation in SSA. However, videoconferencing and TV/radio were added by relevant stakeholders.

Important factors for the implementation relate to several domains. First within the domain of innovation, factors include effectiveness, advantages, costs and design of the intervention. Second, in the Individual domain, user experience, satisfaction and acceptability play a key role. Third, with the Implementation Process domain, the structured use of an implementation process evaluation framework, appropriate implementation strategies, and consideration of barriers and facilitators during implementation were identified as important.

In comparison to previous literature, this study provides new insights specific to the SSA context. To the best of the authors’ knowledge, this is the first review to both outline the technologies implemented for home-based rehabilitation and identify key factors influencing their implementation in SSA.

Previous reviews analysing the use of rehabilitation technologies more broadly, without limiting the focus to SSA, indicated that in addition to the technologies identified in this study, advanced technologies such as robotics and virtual/augmented reality are frequently utilized [22]. One reason for this might be that most published studies in this review were conducted in high- income countries. The needs and challenges in SSA are very different from those in high-income countries. Low-tech solutions are often used in SSA as they offer benefits adapted to the available resources. Low-tech solutions can facilitate access to rehabilitation, for example, by eliminating the need for an internet connection when using SMS or special knowledge for using high-tech solutions.

We identified several key factors relevant to the implementation of Digital Rehabilitation in SSA. These include the intervention’s effectiveness, perceived advantages, costs, and design, as well as user experience, satisfaction, and acceptability. In addition, the use of an implementation process evaluation framework, appropriate implementation strategies, and the systematic consideration of barriers and facilitators were found to be critical. Overall, these findings are in accordance with the results reported by Kaboré et al. [68], who identified barriers and facilitators for digital health interventions in low- and middle income countries. The authors also found that the (cost)effectiveness, user benefits, the design of the intervention, acceptability and motivation to use the intervention are facilitators for implementation. Furthermore, identified barriers associated with implementation are infrastructure, equipment, internet, electricity, and the usability of the intervention [68]. Policies and governments should support the development of an appropriate infrastructure, such as the expansion of the internet, the provision of universal electricity, and the affordability of mobile devices. Although some government activities have been undertaken in various countries to expand digitization, implementation varies widely across SSA countries [69].

We identified relevant implementation determinants within the Innovation, Individual, and Implementation Process domains. However, we were unable to extract any determinants related to the Inner Setting or Outer Setting domains [37]. The Inner Setting domain includes, among other factors, organizational culture, work and physical infrastructure, implementation funding, and intervention delivery. The Outer Setting domain includes policies and legislation as well as local conditions relevant to the implementation of home-based Digital Rehabilitation. This suggests that the introduction of digital home-based rehabilitation in SSA is still in its infancy and has not yet been fully implemented. Although several studies assessed intervention costs or cost-effectiveness [47, 51, 53, 64] none explicitly described or evaluated a reimbursement mechanism for sustaining the intervention beyond the study period. Some studies addressed privacy, anonymity, or confidentiality at the intervention level [46, 48, 49, 62]; however, none of the included studies explicitly evaluated data protection legislation or broader legal and regulatory frameworks relevant to the sustained delivery of home-based Digital Rehabilitation. Furthermore, the included studies provided little or no information on professional or discipline-specific guidelines supporting the implementation or delivery of the interventions. Consideration of these organizational and system-level conditions is essential to support the sustainable implementation of home-based Digital Rehabilitation and enable countries in sub-Saharan Africa to realize its potential benefits [68]. We therefore recommend that future studies explicitly plan, implement, and evaluate determinants across all relevant CFIR domains to generate the context-specific evidence required for long-term implementation.

The absence of findings related to the Inner and Outer Settings also reflects the nature of the implementation strategies employed. Frequently, non-sustainable approaches were utilized, such as offering financial incentives for study participation or providing clients with the necessary devices and internet access to carry out the intervention. However, once the study concludes, there is a significant likelihood that clients will be unable to continue the intervention due to the discontinuation of incentives or resources.

These practices reveal two broader issues relevant to long-term success. First, society needs to be better informed about certain diseases and the benefits of (digital) rehabilitation in general, to better assess the benefits for (home-based) Digital Rehabilitation themselves. Perceived usefulness also has an enormous impact on the motivation to use the intervention [70]. For example, a review by Boateng et al. [71] showed that people in SSA have little knowledge about the risks of hypertension, smoking, physical inactivity, diabetes, stress and excessive alcohol consumption. Public health campaigns disseminated through mass media such as radio, are needed to address this problem, and to inform about public health diseases as also emphasized by the stakeholders. This statement could be underlined by the literature [72]. Leonard et al. [73] further investigated that dissemination, which includes awareness of the innovation, contributes to the success of implementation. Second, the lack of digital device ownership underscores the need to increase the affordability of digital devices and the usage of existing infrastructure, such as using computers available to clients within their community. A review [74] that examined the sustainability of donor-funded health programs beyond the funding lifecycle in Africa, explicitly mentioned this strategy as being conducive for implementation.

Training and capacity-building are also crucial elements that were rarely addressed. We identified only two studies [24, 42] using an implementation strategy that includes training of the healthcare professionals to deliver the intervention. In contrast, several reviews considered this strategy as an important factor for a successful implementation of health interventions [68, 73, 75] although this was not implemented. One reason for this may be that implementation strategies were chosen in which the intervention was not delivered by healthcare professionals, but was outsourced and centralized, and study staff were responsible for delivering the intervention. In addition, the included studies often used low-tech solutions that are relatively easy to use and do not require additional training of healthcare professionals. However, stakeholders mentioned that governments should provide funding and resources for training programs focused on digital health literacy for healthcare professionals and clients to bridge this gap.

The role of implementation frameworks merits further attention. Another finding was that only one study [24] used implementation frameworks to guide the implementation process. The low use of implementation frameworks seen in the included studies contrasts with the findings of Pearce et al. [75], who reported that 52% of the included studies used a framework for the implementation of digital health interventions. The use of one or more implementation frameworks [76] is essential, despite their known limitations [77]. These frameworks can guide the design and execution of studies and inform both the theoretical and empirical perspectives of research teams. Additionally, they support the interpretation of results and help translate knowledge from research into practice. For practitioners, they can provide a common language to familiarize stakeholders with implementation and serve as practical tools for planning, implementing and evaluating implementation interventions in practice [76]. One possible explanation for the limited use of implementation frameworks is that only one study focused on the implementation of the intervention [24]. Most of the included studies in this review [43, 44, 46, 47, 50, 52, 54, 56, 57, 60, 61] focused exclusively on the effectiveness of the intervention without including the implementation of the intervention. Although effectiveness is a key motivator for implementation, implementation and intervention effectiveness should be considered as parts of a single system and not evaluated on their own. All causal mechanisms linking the different system components (intervention and implementation) need to be considered. Otherwise, this represents a significant gap in the research. The accumulation of knowledge in implementation research requires methods of causal inference. These methods must account for the complex structure of the systems in which interventions are implemented. They should also examine how implementation processes actually function [78, 79].

From a health perspective, digital interventions have been applied across a range of conditions in SSA. According to the findings of this review, digital interventions for rehabilitation are being used for people living with HIV, tuberculosis, malaria, depression, stroke, diabetes, hearing loss, and amputation. Three of the communicable diseases that cause a high burden of disease in SSA are HIV, malaria and tuberculosis [80]. Therefore, it is unsurprising that digital interventions have been adopted for these conditions. Access to rehabilitation services in this region is generally limited. As a result, many people do not receive the rehabilitation they need. It is hypothesized that the integration of digital tools will offer a potential solution. The results also show that digital interventions are also frequently used for people with depression. Available evidence suggests that the prevalence of depressive and common mental disorders in the general population is comparable to rates reported in America and Europe [81]. Additionally, these disorders are particularly prevalent among adolescents in SSA [82]. It is reasonable to hypothesize that this target group in particular benefits from digital interventions. As Naslund et al. [83] showed in their narrative review, digital interventions may support the identification of depressive symptoms, improve adherence, and reduce associated symptoms.

Finally, a geographical analysis of the literature reveals limited country coverage. Our results indicate that research regarding the implementation of home-based Digital Rehabilitation has been conducted in only 10 out of 49 countries in the SSA region. One reason for this could be that implementation research has not been conducted outside South Africa, Nigeria, Kenya, Tanzania, Uganda, Rwanda, Cameroon, Zambia, Ethiopia and Ghana and that those countries are pioneers in this field. A similar pattern of results was obtained in the reviews by Kaboré et al. and Ilesanmi & Afolabi [68, 74]. Beyond the findings of this study, stakeholders designing digital interventions for countries other than those mentioned above should consider country-specific religion, culture, policies, regulations, legislation, and infrastructure. The results of this study cannot be extrapolated to all other SSA countries without further verification.

### Limitations

While this study contributes to the understanding of technologies currently utilized for home-based Digital Rehabilitation in SSA and highlights key factors to consider for scaling up Digital Rehabilitation in this region, it also has several limitations that should be acknowledged.

By focusing on studies that examined already implemented digital interventions, we may have missed digital technologies that are still under development and have not yet been implemented. It is possible that articles evaluating the usability of technologies or stakeholder acceptance/readiness to use digital technologies in rehabilitation exist but were not included in our review due to the nature of the search strategy. Consequently, the results may not provide a comprehensive picture of the existing digital technologies used in rehabilitation in SSA.

The inclusion of only a small group of stakeholders with similar backgrounds represents an additional limitation, as other perspectives may not have been captured. We mainly included former practitioners who are currently working in higher education. This could have led to a bias in our results without considering voices from the practical field.

## Conclusion

The findings show that multiple low-tech solutions such as apps, web-based programs, videoconferencing, TV/radio, social media and SMS are already used for home-based rehabilitation in SSA. In addition to their effects on various health outcomes, the findings indicate that home-based Digital Rehabilitation may improve access to healthcare, treatment retention, continuity of care, reduce costs, and reduce stigmatization.

Key factors for successful implementation in SSA are diverse and span several dimensions. These include the effectiveness, advantages, costs, and design of the intervention. Additionally, user experience, satisfaction, and acceptability play a critical role. Further important aspects are the application of an implementation process evaluation framework and the use of appropriate implementation strategies. Finally, careful consideration of barriers and facilitators during the implementation process is essential. To fully realize the potential of home-based Digital Rehabilitation in SSA, factors related to the Inner and Outer Setting domains should also be considered, including structural characteristics, religious and cultural factors, local conditions, politics and legislation, and intervention financing.

The results of this study suggest the development of sustainable approaches, considering all domains of the CFIR 2.0, to bring changes at the systemic level, such as the implementation of an appropriate infrastructure and reimbursement mechanism and the expansion of (digital) health literacy in society.

When designing and implementing home-based Digital Rehabilitation interventions, the resources and challenges of the individual countries in SSA should be examined and considered.

## Supplementary Information

Supplementary Material 1.

Supplementary Material 2.

Supplementary Material 3.

## Data Availability

All data generated or analysed during this study are included in this published article and its supplementary information files.

## Abbreviations

App: Application
CFIR: Consolidated Framework for Implementation Research
GDB: Gross domestic product
HIV: Human immunodeficiency virus
i-PARIHS: Promoting Action on Research Implementation in Health Services
MMAT: Mixed Methods Appraisal Tool
MRC: Medical Research Council
OSF: Open Science Framework
PRISMA-ScR: Preferred Reporting Items for Systematic Reviews and Meta-Analyses extension for Scoping Reviews
RADIC: Rehabilitation for all through Digital Innovations and Competencies
RCT: Randomized controlled trial
RoB 2: Risk of Bias tool 2
RQ: Research Question
SMS: Short-message-services
SSA: sub-Saharan Africa
ST: Stakeholder
TV: Television
