# Supplementary material for: Implementation of home-based Digital Rehabilitation in sub-Saharan Africa: a scoping review
Source: BMC Public Health. 2026 Nov 6;26:3254. doi: 10.1186/s12889-026-30180-8 (PMC13741725; doi:10.1186/s12889-026-30180-8)
Supplement: Supplementary file 2 [file 12889_2026_30180_MOESM2_ESM.docx]

# Appendix 2: Search strategy

**PubMed**

\# 1: **artificial intelligence* [tiab] OR application* [tiab] OR augmented reality [tiab] OR brain computer interface[tiab] OR connected health [tiab] OR computer-based [tiab] OR computer-assisted [tiab] OR computer-aided [tiab] OR computer-supported [tiab] OR computerbased [tiab] OR computerassisted [tiab] OR computeraided [tiab] OR computersupported [tiab] OR digital [tiab] OR ehealth[tiab] OR e-health[tiab] OR electronic health[tiab] OR e-diagnosis[tiab] OR e-intervention[tiab] OR "e-therapy"[tiab] OR exergame [tiab] OR exoskeleton [tiab] OR high-tech medical technology[tiab] OR human-computer interface[tiab] OR hybrid[tiab] OR "information and communication technology"[tiab] OR "Information Technology"[Mesh] OR internet-assisted[tiab] OR internetassisted[tiab] OR internet-based[tiab] OR internet-supported [tiab] OR internetbased[tiab] OR internetaided[tiab] OR internet-aided[tiab] OR "internet of things"[MeSH] OR "internet of things" [tiab] OR machine learning [tiab] OR mhealth[tiab] OR m-health[tiab] OR "mixed reality" [tiab] OR mobile health[tiab] OR "Mobile Applications"[Mesh] OR mobile[tiab] OR remote[tiab] OR robot*[tiab] OR "Robotics"[Mesh] OR telerobot*[tiab] OR tele-robot*[tiab] OR sensors [tiab] OR serious game[tiab] OR serious gaming[tiab] OR smartphone*[tiab] OR tablet-based [tiab] OR tablet-supported [tiab] OR tablet-assisted [tiab] OR tablet based [tiab] OR tablet supported [tiab] OR tablet assisted [tiab] OR technolog*[tiab] OR tele-technolog*[tiab] OR tele-diagnosis[tiab] OR tele-care[tiab] OR "Telerehabilitation"[Mesh] OR tele-rehabilitation[tiab] OR "Telemedicine"[Mesh] OR tele health[tiab] OR tele medicine[tiab] OR telecare[tiab] OR teleconsultation[tiab] OR telediagnosis[tiab] OR telehealth[tiab] OR teletechnology[tiab] OR "Telemetry"[Mesh] OR telemetry[tiab] OR telemonitor*[tiab] OR teletherapy[tiab] OR televideo[tiab] OR "Therapy, Computer-Assisted"[Mesh] OR "User-Computer Interface"[Mesh] OR video-consultation* [tiab] OR videoconferencing[tiab] OR "Videoconferencing"[Mesh] OR "Video Games"[Mesh] OR video game [tiab] OR video gaming [tiab] OR videogame[tiab] OR videogaming [tiab] OR "Video Games"[Mesh] OR video-teleconference[tiab] OR videoteleconference[tiab] OR virtual*[tiab] OR wearable[tiab] OR web-based[tiab] OR web-assisted[tiab] OR "web-supported"[tiab] OR webbased[tiab] OR webassisted[tiab] OR websupported[tiab]**

**#2: "sub saharan africa"[Title/Abstract] OR "sub saharan africa"[Title/Abstract] OR "sub-Sahara"[Title/Abstract] OR "sub-Sahara"[Title/Abstract] OR "SSA"[Title/Abstract] OR "Africa South of the Sahara"[Title/Abstract] OR "Africa South of the Sahara"[Mesh] OR central Africa [tiab] OR East Africa[tiab] OR Eastern Africa[tiab] OR Southern Africa[tiab] OR West Africa[tiab] OR Western Africa[tiab] OR "Africa, Central"[Mesh] OR "Africa, Eastern"[Mesh] OR "Africa, Southern"[Mesh] OR "Africa, Western"[Mesh]**

#3: **((Angola [Title/Abstract] OR Benin[Title/Abstract] OR Botswana[Title/Abstract] OR Burkina Faso[Title/Abstract] OR Burundi[Title/Abstract] OR Cameroon[Title/Abstract] OR Cape Verde[Title/Abstract] OR Central African[Title/Abstract] OR Dem. Rep. Congo[Title/Abstract] OR Ivory Coast[Title/Abstract] OR Djibouti[Title/Abstract] OR Eritrea[Title/Abstract] OR Eswatini [Title/Abstract] OR Ethiopia[Title/Abstract] OR Gabon[Title/Abstract] OR Gambia[Title/Abstract] OR Ghana[Title/Abstract] OR Guinea[Title/Abstract] OR Guinea Equatorial[Title/Abstract] OR Guinea-Bissau[Title/Abstract] OR Kenya[Title/Abstract] OR Lesotho[Title/Abstract] OR Liberia[Title/Abstract] OR Madagascar[Title/Abstract] OR Malawi[Title/Abstract] OR Mali[Title/Abstract] OR Mauritania[Title/Abstract] OR Mauritius[Title/Abstract] OR Mayotte[Title/Abstract] OR Mozambique[Title/Abstract] OR Namibia[Title/Abstract] OR Niger[Title/Abstract] OR Nigeria[Title/Abstract] OR Rwanda[Title/Abstract] OR São Tomé Príncipe[Title/Abstract] OR Senegal[Title/Abstract] OR Seychelles[Title/Abstract] OR Sierra Leone[Title/Abstract] OR Somalia[Title/Abstract] OR South Africa[Title/Abstract] OR South Sudan[Title/Abstract] OR Sudan[Title/Abstract] OR Tanzania[Title/Abstract] OR Togo[Title/Abstract] OR Uganda[Title/Abstract] OR Zambia[Title/Abstract] OR Zimbabwe[Title/Abstract]))**

**#4: #2 OR #3**

#5: **(((((((((((("rehabilitation" [mesh]) OR ("Psychiatric Rehabilitation" [mesh])) OR ("Rehabilitation Nursing" [mesh])) OR ("Rehabilitation Centers" [mesh])) OR ("Neurological Rehabilitation" [mesh])) OR ("Hospitals, Rehabilitation"[Mesh])) OR ("Rehabilitation, Vocational"[Mesh])) OR ("Mouth Rehabilitation"[Mesh])) OR ("Stroke Rehabilitation"[Mesh])) OR ("Rehabilitation Research"[Mesh])) OR ("Physical and Rehabilitation Medicine"[Mesh])) OR ("Rehabilitation of Speech and Language Disorders"[Mesh])) OR ("Correction of Hearing Impairment"[Mesh])**

**#6: ((((((("Physical Therapy Modalities"[Mesh] OR "Physical Therapists"[Mesh] OR "Physical Therapist Assistants"[Mesh] OR "Physical Therapy Specialty"[Mesh] OR "Postoperative Care"[Mesh]) OR ("Physical Therapy Department, Hospital"[Mesh])) OR ("Exercise Therapy"[Mesh])) OR ("Preoperative Exercise"[Mesh]))) OR ((physical therapy [tiab]) OR (physiotherap*[Title/Abstract] OR exercisetherap*[Title/Abstract] OR exercise therapy[Title/Abstract] OR "exercise intervention"[Title/Abstract] OR "therapeutic exercise"[Title/Abstract])) ) OR (exercise therapy [tiab])) OR (therapeutic exercises [tiab])**

**#7: (("Speech Therapy"[Mesh]) OR ("recreation* therap*"[Title/Abstract] OR "speech therap*"[Title/Abstract])) OR ("Language Therapy"[Mesh])**

**#8: ("Occupational Therapy"[Mesh] OR "Occupational Therapy Department, Hospital"[Mesh] OR "Occupational Therapists"[Mesh]) OR (occupation*[Title/Abstract] AND (therap*[Title/Abstract] OR rehabil*[Title/Abstract]))**

**#9: ((("Psychotherapy"[Mesh]) OR (psychotherap*[Title/Abstract])) OR ("psychological therapy"[Title/Abstract])) OR ("psycho-therapy"[Title/Abstract])**

**#10: #5 OR #6 OR #7 OR #8 OR #9**

**#11: #1 AND #4 AND #10**

Filters: **from 2018 – 2023**
