# Supplementary material for: Implementation of home-based Digital Rehabilitation in sub-Saharan Africa: a scoping review
Source: BMC Public Health. 2026 Nov 6;26:3254. doi: 10.1186/s12889-026-30180-8 (PMC13741725; doi:10.1186/s12889-026-30180-8)
Supplement: Supplementary file 3 [file 12889_2026_30180_MOESM3_ESM.docx]

# Appendix 3: Critical appraisal and risk of bias assessment

**Randomized Controlled Trials (RCTs)**

To assess the risk of bias in the selected RCT’s the RoB 2.0 for randomized trials were used. The RoB 2 tool assesses bias in five distinct domains: bias arising from the randomization process (D1), bias due to deviations from intended interventions (D2), bias due to missing outcome data (D3), bias in the measurement of the outcome (D4), and bias in the selection of the reported result (D5). The judgments within each domain lead to an overall risk of bias of: “low risk of bias,” “some concerns,” or “high risk of bias”. If one of the domains are scored with at least one “some concerns” or “high risk” the overall rating can’t be above this rating. The judgment per domain and the overall assessment shows figure 2. Overall the result of RoB assessment was highly impacted by the results of domain 4. This can be explained by the nature of the studies selected, in which participants assessed outcomes in the form of questionnaires and were therefore not blinded to the intervention, which lowered the overall rating of RoB.

Judgement

Low

Some concerns

High

Domains

D1: Bias arising from the randomization process.

D2: Bias due to deviations from intended intervention.

D3: Bias due to missing outcome data.

D4: Bias in measurement of the outcome.

D5: Bias in selection of the reported results.

Figure 2: Results of the risk of bias assessment with the RoB 2.0 of the included RCT's

**Qualitative Studies**

The critical appraisal of the qualitative studies was performed using the JBI Critical Appraisal Checklist for Qualitative Research. The checklist provided a framework for scoring the quality of qualitative studies by addressing different aspects of the research such as ethical considerations, potential bias, integrity of the methodology, and congruity between methods, results and conclusion. The studies were appraised with answering ten quality criteria questions with a “yes”, “no”, “unclear” or “not applicable”. Reviewing the studies, it was discovered that either of the studies did not include statements locating the researchers’ cultural or theoretical position, or the influence of the researcher on the research, which affected to the assessment of determining the level of the quality of the studies. Both selected studies were assessed to be of high quality as the score ranged between 7 and 8 on the JBI Critical Appraisal Checklist for Qualitative Research.

Table 5: Results of the JBI Critical Appraisal Checklist for Qualitative Research of the included qualitative studies

| Questions | Gericke et al. (2021) | Chory et al. (2021) |
| --- | --- | --- |
| **Q1.** | Y | Y |
| **Q2.** | Y | Y |
| **Q3.** | Y | N |
| **Q4.** | Y | Y |
| **Q5.** | Y | Y |
| **Q6.** | N | N |
| **Q7.** | N | N |
| **Q8.** | Y | Y |
| **Q9.** | Y | Y |
| **Q10.** | Y | Y |

**JBI Critical Appraisal Checklist for Qualitative Research items: N = No; U = Unclear; Y = Yes**

Criteria for the critical appraisal of qualitative evidence:

Q1 = Is there congruity between the stated philosophical perspective and the research methodology?

Q2 = Is there congruity between the research methodology and the research question or objectives?

Q3 = Is there congruity between the research methodology and the methods used to collect data?

Q4 = Is there congruity between the research methodology and the representation and analysis of data?

Q5 = Is there congruity between the research methodology and the interpretation of results?

Q6 = Is there a statement locating the researcher culturally or theoretically?

Q7 = Is the influence of the researcher on the research, and vice- versa, addressed?

Q8 = Are participants, and their voices, adequately represented?

Q9 = Is the research ethical according to current criteria or, for recent studies, and is there evidence of ethical approval by an appropriate body?

Q10 = Do the conclusions drawn in the research report flow from the analysis, or interpretation, of the data?

**Mixed Method Studies**

The critical appraisal was performed by using MMAT tool. The MMAT includes five categories of study designs, each with five methodological quality criteria. All studies were rated using the criteria for the mixed methods design. Each criterion was rated as “Yes” if it was met, “No” if it was not met, or “Can’t tell” if insufficient information was available to rate the criteria. This meant that each study received between zero and five “Yes” ratings for each applicable MMAT category. Receiving “Yes” ratings only implies a study has high methodological quality. In line with the MMAT guidance, no overall scores were calculated. In 3/4 of the studies, the reasons for conducting a mixed method study were not clearly explained and the components of the studies were not effectively integrated to answer the research question. In none of the studies, it was described about the interpretation of the combination of the quantitative and qualitative results and the data were analyzed and interpreted separately. In 3/4 studies there was no divergence in the data, or the divergences were addressed properly. Only in one of the studies’ the quality of the qualitative and quantitative components were measured to be high and for the rest of the studies the quality of both components was not possible to be measured as high quality. See all results of the MMAT per study in table 6.

Table 6: Results of the critical appraisal with the Mixed Methods Appraisal Tool (MMAT) of the included mixed methods studies

| Questions | Chory et al. (2022) | Simpson et al. (2021) | Teriö et al. (2019) | Khatib & Hlayisi (2022) |
| --- | --- | --- | --- | --- |
| **Q5.1.** | N | N | N | Y |
| **Q5.2.** | N | N | N | Y |
| **Q5.3.** | N | N | N | N |
| **Q5.4.** | Y | Y | Y | N |
| **Q5.5.** | Y | N | N | N |

**MMAT items: Y= Yes; N = No; N/A = Not applicable in this study; - = Can’t tell based on the article**

Q5.1 = Is there an adequate rationale for using a mixed methods design to address the research question?

Q5.2 = Are the different components of the study effectively integrated to answer the research question?

Q5.3 = Are the outputs of the integration of qualitative and quantitative components adequately interpreted?

Q5.4 = Are divergences and inconsistencies between quantitative and qualitative results adequately addressed?

Q5.5 = Do the different components of the study adhere to the quality criteria of each tradition of the methods involved?
